# Lexical Stress Identification in Cochlear Implant-Simulated Speech by Non-Native Listeners

**DOI:** 10.1177/00238309231222207

**Published:** 2024-01-29

**Authors:** Marita K. Everhardt, Anastasios Sarampalis, Matt Coler, Deniz Bașkent, Wander Lowie

**Affiliations:** Center for Language and Cognition Groningen, University of Groningen, The Netherlands; Department of Otorhinolaryngology/Head and Neck Surgery, University Medical Center Groningen, University of Groningen, The Netherlands; Research School of Behavioural and Cognitive Neurosciences, University of Groningen, The Netherlands; Research School of Behavioural and Cognitive Neurosciences, University of Groningen, The Netherlands; Department of Psychology, University of Groningen, The Netherlands; Research School of Behavioural and Cognitive Neurosciences, University of Groningen, The Netherlands; Campus Fryslân, University of Groningen, The Netherlands; Department of Otorhinolaryngology/Head and Neck Surgery, University Medical Center Groningen, University of Groningen, The Netherlands; Research School of Behavioural and Cognitive Neurosciences, University of Groningen, The Netherlands; W. J. Kolff Institute for Biomedical Engineering and Materials Science, University Medical Center Groningen, University of Groningen, The Netherlands; Center for Language and Cognition Groningen, University of Groningen, The Netherlands; Research School of Behavioural and Cognitive Neurosciences, University of Groningen, The Netherlands

**Keywords:** Cochlear implant simulations, lexical stress, non-native, cue weighting, prosody

## Abstract

This study investigates whether a presumed difference in the perceptibility of cues to lexical stress in spectro-temporally degraded simulated cochlear implant (CI) speech affects how listeners weight these cues during a lexical stress identification task, specifically in their non-native language. Previous research suggests that in English, listeners predominantly rely on a reduction in vowel quality as a cue to lexical stress. In Dutch, changes in the fundamental frequency (F0) contour seem to have a greater functional weight than the vowel quality contrast. Generally, non-native listeners use the cue-weighting strategies from their native language in the non-native language. Moreover, few studies have suggested that these cues to lexical stress are differently perceptible in spectro-temporally degraded electric hearing, as CI users appear to make more effective use of changes in vowel quality than of changes in the F0 contour as cues to linguistic phenomena. In this study, native Dutch learners of English identified stressed syllables in CI-simulated and non-CI-simulated Dutch and English words that contained changes in the F0 contour and vowel quality as cues to lexical stress. The results indicate that neither the cue-weighting strategies in the native language nor in the non-native language are influenced by the perceptibility of cues in the spectro-temporally degraded speech signal. These results are in contrast to our expectations based on previous research and support the idea that cue weighting is a flexible and transferable process.

## 1 Introduction

Cochlear implants (CIs) are auditory prostheses that can partially restore hearing in individuals with profound sensorineural hearing loss. An electrode array inserted in the cochlea sends electrical signals to the auditory nerve directly and by doing so it takes over the functioning of the damaged inner workings of the ear. This electric stimulation makes speech perception possible, yet a speech signal transmitted this way in electric hearing has much less fine spectro-temporal detail than an acoustic speech signal transmitted in normal hearing (for details on electric hearing, see Bașkent et al., 2016). The spectro-temporal degradation obscures those qualities of the speech signal that function as cues to linguistic phenomena (Moberly et al., 2021; Pisoni, 2005; Shannon et al., 2004). This is particularly the case for fundamental frequency (F0), a cue mainly related to the pitch of a speaker’s voice and, in a broader sense, to prosodic features of speech (Chatterjee & Peng, 2008; Gaudrain & Başkent, 2018). Limited access to the prosodic cue F0 in electric hearing has been shown to compromise the ability of listeners to recognize prosodic patterns in the native language (for an overview, see Everhardt et al., 2020). Far less research has been done on the influence of electric hearing on perceptual abilities in a non-native language. That is, there have only been a handful of studies that have looked at more generic non-native listening skills in CI users (e.g., Beeres-Scheenstra et al., 2017, 2020) and the literature on the influence of electric hearing on non-native prosody perception specifically is even more limited. The present study aims to fill that gap, as it explores how spectro-temporal degradations similar to those that can occur in electric hearing through a CI simulation (i.e., an acoustic approximation of electric hearing implemented by a noise-band vocoder) influences the recognition of prosodic patterns—specifically the identification of lexical stress—in a non-native language.

Previous research shows that listeners make sense of a speech signal through the use of multiple cues that are simultaneously available in a speech signal, but that they may weight these cues differently. This cue-weighting theory of speech perception proposes that listeners weight the cues as a function of how informative each cue is for signaling a linguistically meaningful contrast and that listeners attach the greatest functional weight to the most informative and reliable cues for a specific situation (Francis et al., 2000; Francis & Nusbaum, 2002; Holt & Lotto, 2006). For example, cue-weighting comes into play during the perception of lexical stress contrasts where differences in the pattern of stressed and unstressed syllables can have a lexically distinctive function (e.g., *contract* vs. *contract*, where underlined syllables are stressed). In lexical-stress languages (e.g., English and Dutch), stressed syllables are realized with localized phonetic prominence relative to unstressed syllables, but the acoustic correlates of lexical stress differ among these languages (Cole, 2015; Cutler et al., 1997; Fry, 1958; Gay, 1978; Gussenhoven, 2004; Ladd, 2008). In English, the most important acoustic correlate is in the segmental domain. That is, words with initial stress and thus a strong-weak (SW) pattern have a full vowel in the initial (strong) syllable and show a vowel formant structure that is indicative of a peripheral position in the vowel space. Words with stress on the second syllable and thus a weak-strong (WS) pattern, however, have a more centralized vowel in the initial (weak) syllable; the vowel is usually reduced to a schwa-like vowel (Cole, 2015; Cutler, 1986, 2009; Cutler & van Donselaar, 2001; Fear et al., 1995; Fry, 1958; Gay, 1978; Ladd, 2008). In Dutch, another lexical-stress language, the vowel quality contrast is not as strong of a correlate, as vowels in initial weak syllables are hardly ever reduced in Dutch. Instead, acoustic correlates from the prosodic domain, such as variation in F0, intensity, and duration, are more important (e.g., Cutler & van Donselaar, 2001; Sluijter & van Heuven, 1996).

Speech perception studies have shown that listeners can identify a given syllable of a word as stressed depending on its perceived prominence relative to another syllable within that word that is not stressed. Moreover, listeners from different language backgrounds differ in the relative weight they attach to cues to lexical stress in their native language (e.g., Bond & Small, 1983; Cooper et al., 2002; Cutler, 1986, 2009; Cutler & van Donselaar, 2001; Fear et al., 1995; Small et al., 1988; Tremblay et al., 2021; van Heuven & de Jonge, 2011). In these studies, it was shown that native English listeners predominantly rely on the vowel quality contrast. In Dutch, however, prosodic cues have a greater functional weight than vowel quality. This is in line with the cue-weighting theory of speech perception (Francis et al., 2000; Francis & Nusbaum, 2002; Holt & Lotto, 2006), as listeners direct their attention to the most informative cues, which in this case is the most important acoustic correlate of lexical stress in each language. Conversely, perception studies with non-native listeners have shown that listeners generally use the cue-weighting strategies of their native language in other languages, even when another cue might be more informative or reliable in the non-native language (Francis et al., 2000; Francis & Nusbaum, 2002; Holt & Lotto, 2006; Ingvalson et al., 2012; Iverson et al., 2003; Qin et al., 2017; Tremblay et al., 2021; Zhang & Francis, 2010).

For example, Cooper et al. (2002) showed that, compared with native English listeners, native Dutch listeners made greater use of prosodic cues to lexical stress during a forced choice identification task in English where they had to indicate whether initial syllables belonged to a word with initial stress or to a word with an unstressed initial syllable. Similarly, in the study by Fear et al. (1995), native English listeners performed an acceptability-rating task of cross-spliced English words. The vowels in the initial syllable of these words were interchanged with vowels that either matched or mismatched the original vowel in terms of the prosodic cues and the segmental cues to stress, creating four conditions. The results showed that the acceptability ratings could mainly be attributed to differences in vowel quality. This acceptability-rating task was repeated with native Dutch listeners in a follow-up study by Cutler (2009). Compared with the native English listeners in the study by Fear et al. (1995), the Dutch listeners in this study were found to show greater sensitivity to prosodic cues to lexical stress in English. Furthermore, a recent study by Tremblay et al. (2021) that looked into the cue-weighting strategies of native Dutch listeners identifying stressed syllables in English words showed that the Dutch listeners relied more on changes in the F0 contour as a cue to lexical stress in English than the native English listeners. Together these studies show that, despite the fact that the vowel quality contrast is the most important acoustic correlate of lexical stress in English (e.g., Sluijter & van Heuven, 1996), native Dutch learners of English still heavily rely on changes in the F0 contour as a cue to lexical stress in English. What remains unclear is whether a change in the transmission quality of cues in a speech signal plays any role, raising the question of how spectro-temporal degradations similar to those that can occur in electric hearing could affect cue-weighting strategies in different languages, specifically for non-native language learners.

Little is known about how cue-weighting strategies in different languages may be affected by the spectro-temporal degradation of electric hearing, especially when the cue that listeners are most likely to rely on becomes less reliable as a result of this degradation. A number of studies have, however, shed light on the perceptibility of cues in electric hearing that may be of importance to non-native language learners for the perception of lexical stress. Overall, studies with native listeners have demonstrated that typical CI users (i.e., postlingually deafened and implanted adults) identify members of lexical stress pairs less accurately than their normal-hearing (NH) peers when the most important acoustic correlates of lexical stress of that language are in the prosodic domain, particularly in the case of F0 contours (e.g., Morris et al., 2013). Comparable results were found for early deafened and early implanted children (e.g., Lyxell et al., 2009). Other studies have shown similar results for various prosody perception tasks involving changes in F0 contours. For example, Luo et al. (2009) showed that typical CI users and prelingually deafened adolescent CI users identified lexical tone less accurately than NH controls. Similarly, van Zyl and Hanekom (2013) showed that prelingually and postlingually deafened adult CI users were less accurate in distinguishing between questions and statements than their NH peers. Yet, trade-off relationships between F0 cues and covarying acoustic cues can also occur in spectro-temporally degraded conditions (Peng et al., 2012) and CI users, similar to NH listeners, can adjust perceptual weights and somewhat compensate for difficulties in perceiving F0 contours by making use of more informative and reliable cues (Richter & Chatterjee, 2021). The studies by Luo et al. (2009) and van Zyl and Hanekom (2013) also demonstrated that, in some specific situations, CI users could more easily differentiate between vowel quality contrasts than between F0-related contrasts, even though both tasks in general are difficult for CI users. That is, Luo et al. (2009) showed that for a closed set of four vowels differing in vowel formant structure each produced with four lexical tones, the CI users could more accurately identify the vowel category than the lexical tone of these vowels produced in isolation. Similarly, van Zyl and Hanekom (2013) revealed that, when the same speech stimuli from the same speakers were used, the CI users could more accurately differentiate between word pairs with different vowels than differentiate between words produced either as a question or as a statement, indicating that differences in vowel formant structure were more perceptible for these listeners than differences in the F0 contour for these stimuli. These studies thus suggest that, even if both cues are degraded in electric hearing and therefore difficult to perceive, CI users can make more effective use of changes in vowel quality than of changes in the F0 contour as cues to linguistic phenomena.

The present study assesses how, if at all, the presumed difference in perceptibility of cues in spectro-temporally degraded simulated electric hearing affects cue-weighting strategies, specifically in the non-native language. That is, we investigate how native Dutch learners of English weight changes in the F0 contour and changes in vowel quality as cues to lexical stress when identifying stressed syllables in CI-simulated English words and how this compares to the cue-weighting strategies during the identification of stressed syllables in non-CI-simulated English words as well as CI-simulated and non-CI-simulated Dutch words. In the non-CI-simulated condition, the native Dutch learners of English will likely make effective use of changes in the F0 contour as a cue to lexical stress in both Dutch and English since native Dutch listeners generally attach the greatest functional weight to prosodic cues (Cooper et al., 2002; Cutler, 2009; Fear et al., 1995; Tremblay et al., 2021). In the CI-simulated condition, the native Dutch learners of English may potentially re-weight cues and attach relatively more weight to vowel quality changes, since changes in vowel quality could be more perceptible than changes in the F0 contour in spectro-temporally degraded electric hearing, as claimed by few studies with actual CI users in specific situations (Luo et al., 2009; van Zyl & Hanekom, 2013). Here, we assume that the CI simulation in this study could also capture this effect. The question is whether this relative re-weighting of cues to lexical stress in the CI condition will differ between Dutch and English, as the interaction between cue perceptibility and cue weighting in the native and the non-native language has—to our knowledge— never been studied before. Since changes in vowel quality as a cue to lexical stress is more prominent in English to begin with (e.g., Sluijter & van Heuven, 1996), it is likely that the potential re-weighting of cues as a result of the spectro-temporal degradation will be more prominent in English (vs. Dutch).

## 2 Method

### 2.1 Participants

The native Dutch learners of English that took part in the study were divided into two groups, based on their age and experience with English learning in a school setting. Note that these two groups of participants were included in this study as the study is part of a larger project in which we look at differences between younger non-native language learners with relatively limited experience and older non-native language learners with more experience. Although this is not the main focus of this study, including both participant groups did facilitate a simple investigation into the generalizability of our findings to a larger population with individuals differing in age and experience with non-native language learning. As such, the group distinction will be considered in the analysis and discussion (see below).

The two groups of native Dutch participants of this study included twenty-one 12- to 14-year-old adolescent first-year secondary school students (mean age: 12.5 ± 0.28) from a secondary school in the North of the Netherlands (also reported in Everhardt et al., 2019) and thirty-eight 18- to 26-year-old adult first-year students (mean age: 20.7 ± 1.81) from the Department of Psychology of the University of Groningen. The adolescent secondary school students have a limited amount of experience with English learning in a school setting. In terms of the Common European Framework of Reference for Languages (CEFR; Council of Europe, 2020), a standardized measure that organizes language proficiency in six levels from A1 to C2, their estimated proficiency level of English listening skills is A2 (Inspectie van het Onderwijs, 2019). The adult university students have more years of experience with English learning in a school setting and have an estimated CEFR level of at least B2/C1 as they have to meet these English language requirements of pre-university education in order to enter Dutch universities. Both participant groups started learning English as a non-native language when their native language was largely established.

The participants were required to have normal hearing and normal (or corrected to normal) vision. Normal hearing was assessed through pure-tone audiometry at octave frequencies from 250 to 8000 Hz. All adolescents and 35 adults showed audiometric thresholds ⩽20 dB HL at all frequencies tested. Three adult participants showed thresholds ⩽20 dB HL at octave frequencies from 250 to 4000 Hz and thresholds between 20 and 40 dB HL at 8000 Hz. These participants were still included in the study as we assumed that normal-hearing thresholds up to 4000 Hz matters most since most speech energy of the stimuli is in the lower frequency range.

The study and data collection for the adolescent group was approved by the Research Ethics Review Committee of the Faculty of Arts, University of Groningen (CETO 58255883). In order to recruit first-year psychology students, a separate study protocol was necessary. This protocol was approved by the Ethics Committee of Psychology of the University of Groningen (ECP PSY-1819 S-0176). Written informed consent was obtained from all participants and the parents/guardians of the adolescents before participation in the study. The adolescent secondary school students had a chance to win a €25 gift card determined by a raffle. The adult university students received course credit for participation.

### 2.2 Stimuli

Stimuli included 10 English and 10 Dutch lexical stress pairs (see Table 1). The English stimuli were disyllabic lexical stress pairs with the stress either on the first syllable (SW pattern) or on the second syllable (WS pattern) such as *contract*/*contract*. These pairs were selected from the English component of the public web interface of Celex (WebCelex; http://celex.mpi.nl/) and all showed a phonological vowel distinction between strong initial syllables and weak initial syllables. That is, vowels in initial weak syllables were all reduced to schwa. The Dutch stimuli were both disyllabic and trisyllabic lexical stress pairs. Compared with English, Dutch does not have as many lexical stress pairs and even fewer disyllabic stress pairs (Cutler & van Donselaar, 2001). As the number of Dutch disyllabic stress pairs is limited, it was necessary to also include Dutch trisyllabic stress pairs. To ensure comparability between Dutch and English stimuli, trisyllabic pairs could only be included if the stress falls either on the first syllable (strong-weak-weak [SWW] pattern) or on the second syllable (weak-strong-weak [WSW] pattern); trisyllabic pairs where the stress falls on the third syllable (weak-weak-strong [WWS] pattern) for one member of the pair (e.g., *ondergaan*/*ondergaan*) could not be included. The Dutch stimuli were six disyllabic lexical stress pairs and four trisyllabic lexical stress pairs with stress either on the first syllable or on the second syllable such as *voornaam*/*voornaam* and *doorlopen*/*doorlopen*. These pairs were selected from the Dutch component of WebCelex and none showed a phonological vowel distinction between strong initial syllables and weak initial syllables.

**Table 1.** Dutch and English Lexical Stress Pairs Where One Member of a Pair Has Stress on the First Syllable (SW or SWW) and the Other Member of a Pair Has Stress on the Second Syllable (WS or WSW).

	SW(W)		WS(W)	
Dutch	canon	/'kanɔn/	kanon	/ka'nʒn/
	doordringen	/'dordrɪŋən/	doordringen	/dor'drɪŋən/
	doorlopen	/'dorlopən/	doorlopen	/dor'lopən/
	misbruik	/'mɪsbrœyk/	misbruik	/mɪs'brœyk/
	Plato	/'plato/	plateau	/pla'to/
	Servisch	/'sɛrvis/	servies	/sɛr'vis/
	uitstekend	/'œytstekənt/	uitstekend	/œyt'stekənt/
	voorkomen	/'vorkomən/	voorkomen	/vor'komən/
	voornaam	/'vornam/	voornaam	/vor'nam/
	voorspel	/'vorspɛl/	voorspel	/vor'spɛl/
English	conduct	/'kɒndʌkt/	conduct	/kən'dʌkt/
	conflict	/'kɒnflɪkt/	conflict	/kən'flɪkt/
	console	/'kɒnsəʊl/	console	/kən'səʊl/
	construct	/'kɒnstrʌkt/	construct	/kən'strʌkt/
	content	/'kɒntɛnt/	content	/kən'tɛnt/
	contract	/'kɒntrakt/	contract	/kən'trakt/
	convict	/'kɒnvɪkt/	convict	/kən’vɪkt/
	object	/'ɒbdʒɛkt/	object	/əb'dʒɛkt/
	produce	/'prɒdjuːs/	produce	/prə'djuːs/
	subject	/'sʌbdʒɛkt/	subject	/səb'dʒɛkt/

*Note.* Underlined syllables are stressed. SW = strong-weak; SWW = strong-weak-weak; WS = weak-strong; WSW = weak-strong-weak.

The stimuli were recorded by multiple female speakers to ensure speaker variability while keeping the mean F0 of different speakers within a similar range. The English stimuli were recorded by five female adult native speakers of English, two from the United Kingdom who speak with a Standard Southern British English dialect, and three from the United States who speak with a General American dialect. The Dutch stimuli were recorded by five female adult native speakers of Dutch who all speak with a Standard Dutch (Dutch: *Standaardnederlands*) dialect. Each speaker recorded the 10 lexical stress pairs in both forms: with stress on the first syllable and with stress on the second syllable. The speakers were instructed to produce each word at least three times and as naturally as possible. The recording sessions were preceded by practice sessions to ensure naturalness of the produced stimuli and to familiarize the speakers with the stimuli. The stimuli were recorded in a sound-attenuated booth at a sampling frequency of 48 kHz and sampling depth of 16 bit.

For each speaker, the most natural production of each member of a lexical stress pair was selected by a trained linguist (the first author). Selection was based on the auditory naturalness of the stress pattern (e.g., overemphasized stress patterns were not selected). If all productions of a word were produced with a natural stress pattern, selection was based on the clarity of the recording (e.g., no obvious background noise). The 10 lexical stress pairs per language were subsequently evenly divided between the five speakers, such that each native speaker contributed exactly two lexical stress pairs (2 words × 2 stress patterns) to the stimuli set. These 40 stimuli (2 languages × 5 speakers × 2 words × 2 stress patterns) were acoustically analyzed with respect to changes in peak F0, peak intensity, duration, and vowel formant structure between words with a SW pattern and words with a WS pattern (note that SW-WS henceforth refers to both SW-WS word pairs and SWW-WSW word pairs), separately for the first and second syllable. Peak F0, peak intensity, duration and the first three vowel formants (F1, F2, F3) at vowel midpoint were measured in Praat for all first and second syllables (version 6.0.37; Boersma & Weenink, 2018). Changes in peak F0, peak intensity, and duration between strong syllables and their weak counterparts were manually calculated. Euclidean distances in mel-transformed vowel formants between strong and weak syllables were calculated in the R environment using the *dist2* function of the *flexclust* package (version 1.4-1; Leisch et al., 2022). Mean changes and mean Euclidean distances of these cues for each syllable per language are provided in Table 2. Strong syllables had higher peak F0, higher peak intensity, and longer duration than their weak counterparts. The changes in these cues did not significantly differ between Dutch and English for neither the first nor the second syllable (p>.05) . The Euclidean distances show a large difference in vowel formant structure for initial English syllables, which can be explained by the more centralized pronunciation of vowels in weak initial syllables compared with vowels in strong initial syllables in English. Importantly, there was a significant difference between Dutch and English in the Euclidean distance in vowel formants between strong initial syllables and weak initial syllables, t(36)=−6.7,p<.001 .

**Table 2.** Change in Peak F0, Change in Peak Intensity, Change in Duration, and Euclidean Distance in Vowel Formants Between Strong and Weak Syllables for the First and Second Syllable Averaged Across All Words for Dutch and English.

	∆ F0 (semitones)		∆ intensity (dB)		∆ duration (ms)		*d* formants (mel)	
	*M*	*SD*	*M*	*SD*	*M*	*SD*	*M*	*SD*
Dutch								
$\sigma_{1}$	2.29	1.53	5.24	3.68	78.6	44.8	77.6	34.2
$\sigma_{2}$	1.89	3.29	8.16	2.84	45.4	54.7	67.1	27.4
English								
$\sigma_{1}$	2.98	3.52	5.01	4.02	97.3	49.0	237.2	96.2
$\sigma_{2}$	2.81	5.44	5.15	4.48	51.7	44.1	34.4	13.2

*Note.* ∆ = change; *d* = distance; $\sigma_{1}$ = first syllable; $\sigma_{2}$ = second syllable; SD = standard deviation.

**Figure 1.**
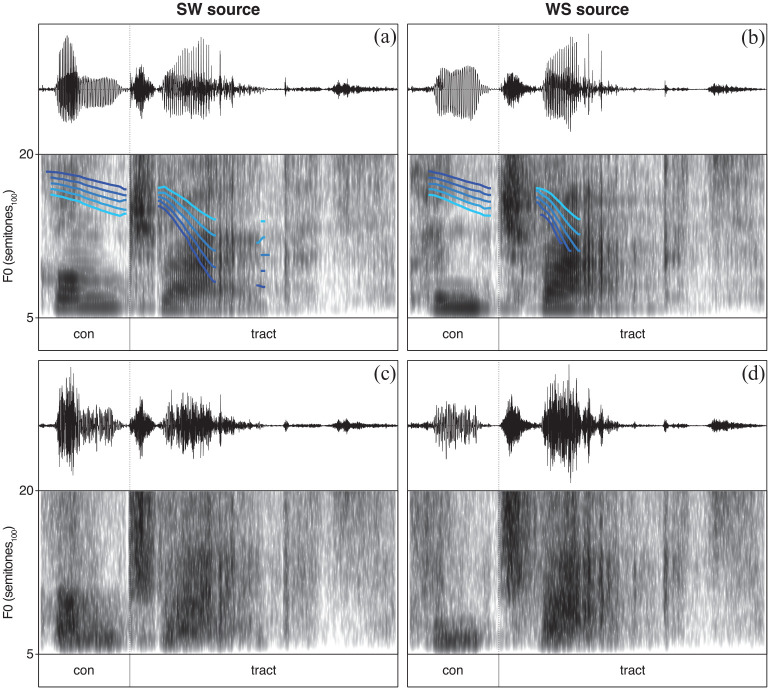
Example stimuli showing the F0 contours and the spectro-temporal detail for the SW source (a) and WS source (b) stimuli and for the subsequently vocoded SW source (c) and WS source (d) stimuli. The 5-step F0 continua range from SW (dark blue line) to WS (light blue line) with three ambiguous steps in between. *Note.* Syllable duration and intensity are neutralized.

The recorded stimuli discussed and analyzed above were manipulated for inclusion in the experiment. The stimuli manipulations focused on changes in the F0 contour and in vowel quality as cues to lexical stress. Changes in intensity and duration as cues to lexical stress are disregarded in this study. As such, the first step in stimuli manipulations involved the neutralization of these latter cues. Duration was neutralized by lengthening or shortening syllables to the average duration of that syllable across stress patterns and intensity was similarly neutralized by increasing or decreasing the root-mean-square (RMS) intensity of syllables to the average RMS intensity of that syllable across stress patterns, making syllable duration and intensity identical for the SW and the WS recordings of a lexical stress pair. Duration and intensity manipulations were carried out in Praat (version 6.0.37; Boersma & Weenink, 2018). While keeping duration and intensity constant, the next step in stimuli manipulations involved the creation of 5-step F0 continua. One end of a continuum (Step 1) represents the SW F0 contour and the other end (Step 5) the WS F0 contour, with three ambiguous steps in between. The F0 manipulations were applied to both the SW and WS source stimuli and were carried out using Pitch-Synchronous Overlap-Add (PSOLA) in Praat (version 6.0.37; Boersma & Weenink, 2018). An example of the 5-step F0 continuum manipulations can be found in Figure 1, which also shows the difference in the spectro-temporal detail between the SW source (Panel A) and WS source (Panel B) stimuli. Finally, acoustic CI simulations of the 200 manipulated stimuli (2 languages × 5 speakers × 2 words × 2 source recordings × 5 F0-continuum steps) were created by means of a vocoder (version 1.0; Gaudrain, 2016) implemented in MATLAB (R2018a). Vocoded stimuli were created using a 6-channel noise-band vocoder with a bandwidth of 250–8700 Hz and Greenwood map, using zero-phase 12th order Butterworth filters with matching analysis and synthesis filters. The temporal envelope was extracted by half-wave rectification and low-pass filtering at a cut-off of 100 Hz using a zero-phase 4th order Butterworth filter. The influence of the CI simulations on the stimuli is visualized in Figure 1, showing the absence of F0 contours and a reduction in spectro-temporal detail for the vocoded SW source (Panel C) and WS source (Panel D) stimuli. Both the unprocessed (i.e., non-vocoded) and vocoded stimuli were included in the final stimuli set, resulting in a total of 400 stimuli (2 languages × 5 speakers × 2 words × 2 source recordings × 5 F0-continuum steps × 2 processing strategies) that were used in the lexical stress identification task discussed below.

### 2.3 Procedure

Participants completed a listening task assessing the influence of the CI simulation on lexical stress identification in Dutch and English words. The experiment was developed and run in OpenSesame (version 3.2.5; Mathôt et al., 2012). Participants performed a single-interval two-alternative forced choice (1I-2AFC) lexical stress identification task with unprocessed and vocoded stimuli. During each trial, they were presented with an auditory stimulus over headphones after which they were asked to indicate whether the first syllable or the second syllable was stressed by pressing the corresponding key. The response options were presented as written text and were accompanied by the corresponding keys (i.e., [*z*] for first syllable, [*m*] for second syllable). The option “first” (Dutch: “eerste”) was always positioned on the left of the screen and the option “second” (Dutch: “tweede”) was always positioned on the right of the screen. The stimuli were presented in randomized order with the constraint that immediate succession of same-word stimuli, regardless of the stress pattern, would not be possible.

The experiment was divided into four blocks, one for each processing condition per language. The blocks were presented in pseudo-randomized order; participants were either presented with the two blocks with Dutch stimuli first and the two blocks with English stimuli next, or vice versa. The order of the processing blocks was also counterbalanced but was identical for both languages. That is, if participants were first presented with the unprocessed block and then with the vocoded block in the two subsequent Dutch blocks, they would also be presented with the unprocessed block first and the vocoded block next in the two subsequent English blocks. Participants were randomly assigned to one of the four possible block orders. Each block started with a practice session of five trials. The stimuli in the practice sessions were randomly selected from a subset of the experimental stimuli. These stimuli were either true SW stimuli (i.e., SW-source stimuli from the end of the F0 continuum that represents the SW F0 contour) or true WS stimuli (i.e., WS-source stimuli from the other end of the F0 continuum that represents the WS F0 contour). During the practice sessions, participants received feedback on the accuracy of their selection of the stressed syllable. In the experiment proper, no feedback was given.

The adolescent participants performed the experiment at their secondary school. Written informed consent was obtained from participants and their parents/guardians at least one day before starting the listening task. Similarly, normal hearing was assessed at least one day before starting the listening task. Audiometric testing was done for each participant individually and also took place at their school. On the day of the listening task, participants received verbal information regarding stress patterns and vocoded speech. The adolescent participants were all already familiar with stress patterns and they were presented with a vocoded speech sample so they could familiarize themselves with vocoded speech. Task-specific instructions were provided in writing during the listening task. They were instructed to pay attention to the stress pattern of the stimuli and to indicate for each stimulus whether the first or the second syllable was stressed as quickly and accurately as possible. They were made aware that they would hear the same word more than once, but that this did not necessarily mean that the stress pattern was the same. They were told that the stimuli were manipulated to reflect the SW and WS stress patterns, meaning that different examples of the same word sometimes reflected the SW stress pattern and sometimes that of the WS stress pattern. In between blocks, participants were allowed to take short breaks. The adolescent participants performed the listening task simultaneously but on individual computers in a classroom setting. The listening task was completed in approximately 45 min.

The adult participants were tested individually in the lab. Written informed consent was obtained in the lab on the day of testing. Similarly, normal hearing was assessed in the lab on the day of testing. Prior to starting the listening task, the adult participants received similar verbal information as the adolescent participants. The adult participants were also all already familiar with stress patterns and they were also presented with a vocoded speech sample so they could familiarize themselves with vocoded speech. The task-specific written instructions were identical for the adolescent and adult participants. The adult participants completed the listening task in approximately 35 min.

## 3 Results

The response data were analyzed by fitting generalized additive mixed-effects models (GAMMs) in the R environment (version 4.2.1) using the *bam* function of the *mgcv* package (version 1.8-40; Wood, 2022). Unlike generalized linear mixed-effects models (GLMMs), GAMMs can fit non-linear response patterns. The lexical stress identification patterns were fitted as the probability of a WS response across the F0 continuum. The need for predictor variables and by-participant and by-stimulus random factor smooths was assessed using binary difference smooths. Given the five steps of the F0 continuum and the fact that the number of basis functions (k) cannot be higher than the number of measurement points of a particular variable, the k-parameter of each smooth was set to 5. Autocorrelation in the residuals of subsequent F0 points in the F0 continuum was controlled for by refitting models with specified *rho* and *AR.start* parameters (Wieling, 2018). The final model included an interaction between *processing* (unprocessed vs. vocoded) and *language* (Dutch vs. English) and an interaction between *source* (SW source vs. WS source) and *group* (non-native adolescents vs. adults). The model also included by-participant factor smooths across the F0 continuum for the interaction between the predictors *processing, source*, and *language* as well as by-stimulus factor smooths across the F0 continuum for the interaction between the predictors *processing, source*, and *group*. Model criticism was applied to the final model by excluding observations with residuals >2.5 SDs from the mean. The nature of the difference (i.e., constant and/or non-linear) for necessary predictor variables was subsequently assessed using ordered factor predictors (Wieling, 2018). The fitted response patterns and difference smooths for significant predictors were visualized using the *plot_smooth* and *plot_diff* functions of the *itsadug* package (version 2.4.1; van Rij et al., 2022). The data and code are available at https://doi.org/10.34894/KEMJYR.

**Figure 2.**
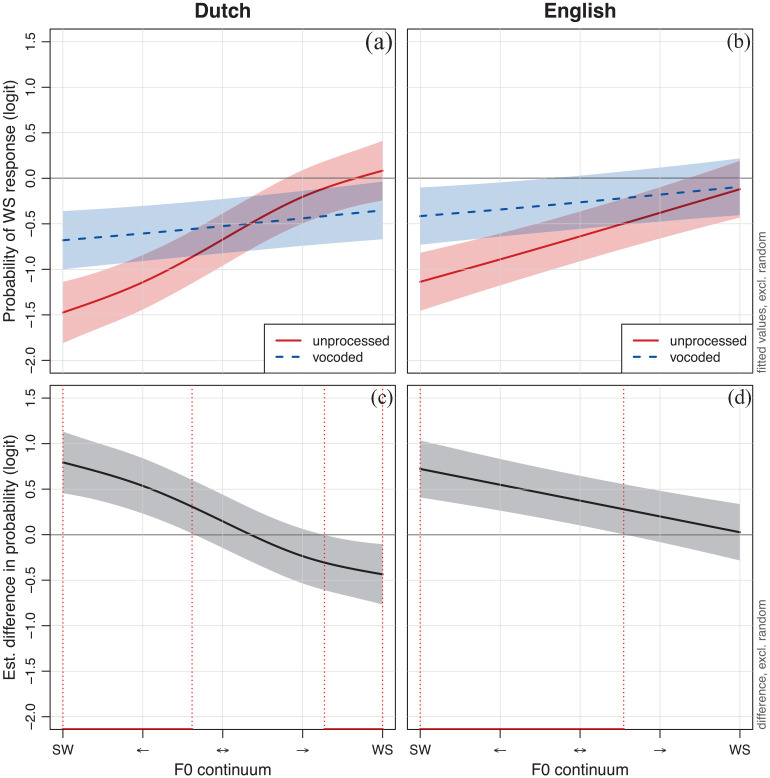
Probability of a WS response across the 5-step F0 continuum for unprocessed (solid red line) and vocoded (dashed blue line) stimuli in Dutch (a) and English (b) as well as the estimated difference in the WS response probability across the 5-step F0 continuum between unprocessed and vocoded stimuli in Dutch (c) and English (d). *Note.* Shaded areas show the 95% confidence bands. Significant differences are marked in red.

The interaction between *processing* and *language* is visualized in Figure 2 (note that the smooths are fitted for the SW source stimuli and the non-native adults, as these are the respective reference levels for the predictors *source* and *group*). The response patterns show that the probability of a WS response increases as the F0 continuum moves from SW to WS, but that the F0 continuum is more effectively utilized in the unprocessed (vs. vocoded) condition, $\chi^{2}=113.93,p<.001$ . The results also revealed a significant non-linear difference in the response patterns between Dutch and English, suggesting a greater sensitivity to F0 contour changes in Dutch (vs. English), $\chi^{2}=20.35,p<.001$ . The significant interaction between *processing* and *language* is mainly due to the fact that the difference smooth (i.e., the difference in response patterns between unprocessed and vocoded stimuli) is significantly more non-linear for Dutch (vs. English), $\chi^{2}=13.06,p<.001$ .

**Figure 3.**
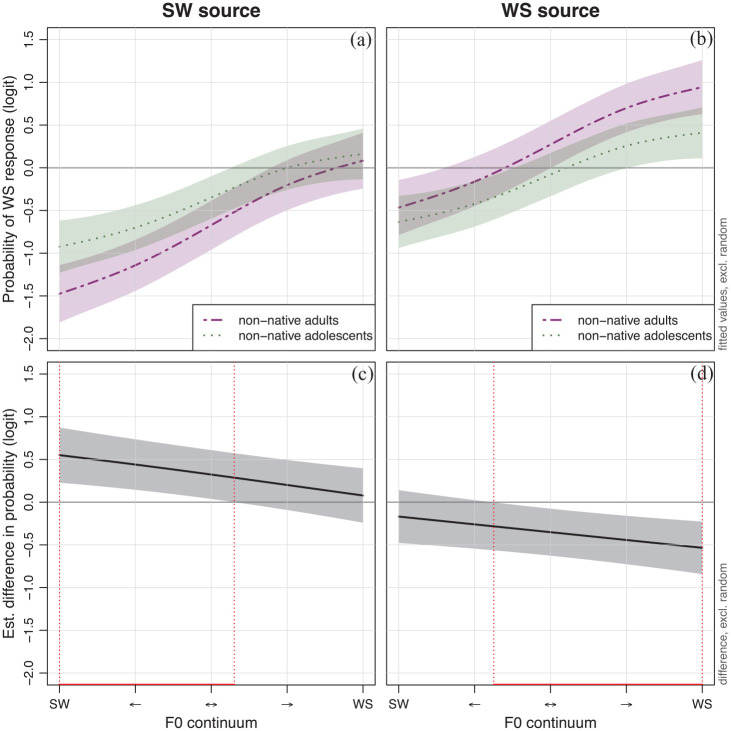
Probability of a WS response across the 5-step F0 continuum for non-native adults (dot-dashed purple line) and adolescents (dotted green line) in SW source (a) and WS source (b) stimuli as well as the estimated difference in the WS response probability across the 5-step F0 continuum between non-native adults adolescents in SW source (c) and WS source (d) stimuli. *Note.* Shaded areas show the 95% confidence bands. Significant differences are marked in red.

Figure 3 shows the interaction between *source* and *group* (note that the smooths are fitted for the unprocessed Dutch stimuli, as these are the respective reference levels for the predictors *processing* and *language*). The response patterns show a non-linear difference in response patterns between the non-native adults and adolescents, which suggests that the adults are more attuned to changes in the F0 contour as a cue to lexical stress than the adolescents, $\chi^{2}=10.16,p=.001$ . The results also revealed a significant constant difference in the response patterns between SW and WS source stimuli, indicated by an overall increase in the probability of a WS response for WS (vs. SW) source stimuli across the entire F0 continuum, z=6.51,p<.001 . The constant difference between SW source and WS source stimuli was in turn significantly greater for the non-native adults (vs. adolescents), z=−5.25,p<.001 .

## 4 Discussion

The present study used a lexical stress identification task to investigate whether the perceptibility of cues to lexical stress in spectro-temporally degraded simulated electric hearing would affect the cue-weighting strategies in Dutch and English for native Dutch learners of English.

Overall, the CI simulation had a significant impact on the lexical stress identification patterns. On the one hand, looking at the cue-weighting strategies in the unprocessed condition, the results showed that the native Dutch learners of English made use of changes in the F0 contour as a cue to lexical stress in both Dutch and English, their native and non-native language respectively. In both languages, they relied on the cue that has a greater functional weight in their native language than in their non-native language (e.g., Bond & Small, 1983; Cooper et al., 2002; Cutler, 1986, 2009; Cutler & van Donselaar, 2001; Fear et al., 1995; Small et al., 1988; Tremblay et al., 2021; van Heuven & de Jonge, 2011). It is possible that this finding is due to the fact that participants were tested in their native and non-native language on the same day, but this potential priming effect was minimized by counterbalancing the block order between participants. On the other hand, looking at the cue-weighting strategies in the vocoded condition, listeners were unable to utilize this cue as effectively. This is unsurprising given the spectro-temporal degradation of the vocoded stimuli (Bașkent et al., 2016; Chatterjee & Peng, 2008; Gaudrain & Başkent, 2018; Pisoni, 2005; Shannon et al., 2004). The lower quality of transmission of F0 in the CI-simulated speech signal likely hindered the utilization of this cue and compromised the identification of the lexical stress pattern across the F0 continuum. An overview of similar findings across different studies can be found in Everhardt et al. (2020), which showed that reduced accuracy in the ability to identify various prosodic patterns in speech can mainly be attributed to the way F0 is encoded in a spectro-temporally degraded speech signal.

The results of the present study also showed that the influence of the CI simulation on the response patterns did not significantly differ between the groups. In other words, both the non-native adolescents and the non-native adults used F0 contour changes in the vocoded condition less effectively than in the unprocessed condition. The adolescents and the adults are all NH listeners with no prior experience with simulated electric hearing, but differ in age (hence, potential developmental effects) and experience with non-native language learning. Important to note is that the non-native adults included in this study did show greater sensitivity to lexical stress cues than the non-native adolescents, as they generally made more effective use of the F0 continuum and also showed a bigger contrast in responses between SW and WS source stimuli. While the findings of some studies suggest that the non-native adolescents of this study are of an age where the understanding of prosodic patterns is expected to be adult-like (e.g., Wells et al., 2004), our results indicate that the prosody perception abilities of the adolescents may not have been completely adult-like but instead will continue to develop over time (e.g., Cutler & Swinney, 1987). Nonetheless, the fact that the non-native adolescents and non-native adults were similarly impacted by the processing condition implies that our results on the influence of electric hearing on lexical stress identification patterns are robust and likely generalizable.

Interestingly, in the unprocessed condition, changes in the F0 contour as a cue to lexical stress was more effectively used in the native language compared with the non-native language. The acoustic analysis (described in the method section above) of the recorded stimuli revealed that changes in peak F0 between strong syllables and their weak counterparts did not significantly differ between the Dutch and English stimuli. This suggests that the difference in the usage of F0 contour changes between these two languages can probably not be attributed to an acoustic difference of this cue in the original speech signal. It is also unlikely that the F0 contour manipulations could explain this difference, as these manipulations are based on the original recordings. As such, the question of why the listeners showed greater sensitivity to F0 contour changes in Dutch than in English remains unclear. Conversely, changes in vowel quality as a cue to lexical stress were not differently used in the native and non-native language. Listeners did not rely more on changes in vowel quality in English than in Dutch even though this cue has a greater functional weight in English (e.g., Bond & Small, 1983; Cooper et al., 2002; Cutler, 1986, 2009; Cutler & van Donselaar, 2001; Fear et al., 1995; Small et al., 1988; Tremblay et al., 2021). That is, the difference in response patterns between words with full initial vowels (SW source) and words with reduced initial vowels (WS source) was similar for Dutch and English. Note that the vowel quality contrast generally is more prominent in English, where vowels in initial weak vowels are more centralized than vowels in initial strong vowels (e.g., Cutler, 1986; Fear et al., 1995). The acoustic analysis (described in the method section above) of the recorded stimuli also showed that the Euclidean distance in vowel formants between strong initial syllables and their weak counterparts significantly differed between the Dutch and English stimuli. It shows that the vowel quality contrast is more prominent in English and that listeners therefore may have attached a greater functional weight to this cue (Francis et al., 2000; Francis & Nusbaum, 2002; Holt & Lotto, 2006). Were this the case, listeners should have re-weighted the cues to lexical stress by increasing the relative weight attached to vowel quality. However, this study did not provide clear evidence for such an increase in the unprocessed condition.

The main aim of this study was to explore how, if at all, a change in cue perceptibility could affect cue weighting, particularly in situations where the cue that listeners are most likely to rely on becomes less reliable and another cue becomes increasingly more reliable as a result of the quality of the speech signal. So far, we have seen that in the vocoded condition the identification of lexical stress across the F0 continuum was compromised in both Dutch and English and that in the unprocessed condition the native Dutch learners of English mainly relied on changes in the F0 contour as a cue to lexical stress in both their native and their non-native language. The question is whether these listeners weighted the cues to lexical stress in the CI-simulated English words similarly to in non-CI-simulated English words or whether they re-weighted these cues in CI-simulated English words by attaching relatively more weight to the presumably more perceptible vowel quality contrast cue, as was claimed by few previous studies. The results revealed no significant interaction between the vowel quality contrast and the processing condition, indicating that listeners generally did not rely more on vowel quality in CI-simulated words. Changes in vowel quality were similarly used in the unprocessed and the vocoded condition. The results also revealed no significant three-way interaction between the vowel quality contrast, the processing condition, and language, which shows that the lack of a difference in the relative weighting of vowel quality as a cue to lexical stress between CI-simulated and non-CI-simulated words also did not differ between Dutch and English. The native Dutch learners of English did not attach relatively more weight to the vowel quality contrast when identifying the stressed syllable in vocoded English words compared with either unprocessed English words or vocoded Dutch words, despite the fact that previous research has implied that this cue is more perceptible than F0 contour changes in electric hearing (Luo et al., 2009; van Zyl & Hanekom, 2013) and also has a greater functional weight in English (e.g., Bond & Small, 1983; Cooper et al., 2002; Cutler, 1986, 2009; Cutler & van Donselaar, 2001; Fear et al., 1995; Small et al., 1988; Tremblay et al., 2021). Contrary to our expectations based on the literature, the change in perceptibility of cues thus did not play any role in the relative re-weighting of cues to lexical stress in the non-native language. Since our observations were similar for the adolescents and adults who differed in both age and developmental stage as well as their experience with non-native language learning, our results seem robust and generalizable.

## 5 Conclusion

This study investigated whether the presumed difference in perceptibility of cues to lexical stress in CI-simulated speech (as was implied by previous research) could affect how native Dutch learners of English would weight these cues in their non-native language. Our test population included non-native adolescents and non-native adults, but no matter their age and experience with non-native language learning, we found no compelling evidence in support of a re-weighting of cues to lexical stress. Listeners did not attach relatively more weight to the vowel quality contrast as a cue to lexical stress when identifying stressed syllables in the CI-simulated English words compared with the non-CI-simulated English words or in the CI-simulated Dutch words, even though this cue was claimed by previous studies to be more perceptible in electric hearing and has a greater functional weight in English. This lack of an interaction between cue perceptibility and cue weighting in the native and the non-native language shows that non-native lexical stress identification in CI-simulated speech is not influenced by a change in the perceptibility of cues to lexical stress.
